# CDC Grand Rounds: Public Health Strategies to Prevent and Treat Strokes

**DOI:** 10.15585/mmwr.mm6618a5

**Published:** 2017-05-12

**Authors:** Mary G. George, Leah Fischer, Walter Koroshetz, Cheryl Bushnell, Michael Frankel, Jennifer Foltz, Phoebe G. Thorpe

**Affiliations:** ^1^Division for Heart Disease and Stroke Prevention, National Center for Chronic Disease Prevention and Health Promotion, CDC; ^2^Division of Preparedness and Emerging Infections, National Center for Emerging and Zoonotic Infectious Diseases, CDC; ^3^National Institute of Neurological Disorders and Stroke, National Institutes of Health, Washington, D.C.; ^4^Wake Forest Baptist Medical Center, Winston-Salem, North Carolina; ^5^Emory University School of Medicine, Atlanta, Georgia; ^6^Office of the Associate Director for Science, CDC.

Worldwide, stroke is the second leading cause of death and a leading cause of serious long-term disability. In the United States, nearly 800,000 strokes occur each year; thus stroke is the fifth leading cause of death overall and the fourth leading cause of death among women (*1*). Major advances in stroke prevention through treatment of known risk factors has led to stroke being considered largely preventable. For example, in the United States, stroke mortality rates have declined 70% over the past 50 years, in large part because of important reductions in hypertension, tobacco smoking, and more recently, increased use of anticoagulation for atrial fibrillation (*2*,*3*). Although the reduction in stroke mortality is recognized as one of the 10 great public health achievements of the 20th century (*4*), gains can still be made. Approximately 80% of strokes could be prevented by screening for and addressing known risks with measures such as improving hypertension control, smoking cessation, diabetes prevention, cholesterol management, increasing use of anticoagulation for atrial fibrillation, and eliminating excessive alcohol consumption (*5*,*6*).

## Risk Factors and Special Populations

Approximately 75% of persons who have a stroke have hypertension, making hypertension the most potent modifiable risk factor for stroke. Hypertension causes weakening of the arteries and can lead to either of the two types of stroke: ischemic stroke and intracerebral hemorrhage. Currently, 29.3% of U.S. adults aged ≥18 years have hypertension, and only about half of these adults have their blood pressure controlled (54%) (*7*). Hypertension is more prevalent among non-Hispanic blacks (blacks) than non-Hispanic whites (whites) (41.2% compared with 28%). Blacks are two to three times more likely to have a stroke than are whites and do so at an earlier age than whites; hypertension is thought to be a more potent risk factor for stroke among blacks and Hispanics than among whites (*8*,*9*). Increasing evidence indicates that hypertension at older ages is an important contributor to vascular cognitive impairment or vascular dementia, which often coexists with other forms of dementia, such as Alzheimer’s disease. Recent studies indicate that hypertension might potentiate the impact of Alzheimer’s disease and its role in causing microbleeds, microinfarcts, white matter disease, and multiple small strokes that might not be clinically noticeable at the time but can lead to dementia, as well as possible involvement in the accumulation of amyloid plaques in the brain (*10*,*11*). Approximately two thirds of adults aged >60 years have hypertension (*12*), putting them at increased risk for both stroke and dementia. Having hypertension in mid-life (ages 45–64 years) is strongly associated with risk for vascular cognitive impairment and dementia later in life (*13*).

Although men have a higher age-adjusted incidence for stroke, women live longer and thus have a higher lifetime risk of stroke than men (*14*). Approximately twice as many women die from stroke than from breast cancer each year (*1*). Approximately 60% of persons who die from stroke are women, and women tend to have worse functional outcomes in terms of returning to baseline activities of daily living and quality of life after experiencing a stroke. Some risk factors that are unique to women include pregnancy, gestational diabetes, eclampsia and preeclampsia, changes in hormonal status, and postmenopausal hormone use. Several studies have shown that having preeclampsia or gestational hypertension increases a woman’s risk for stroke approximately twofold, and women who experience preeclampsia have a fourfold increased risk for developing future hypertension (*14*). Among women who use oral contraceptives, obesity and hypercholesterolemia increase the risk for stroke 4.6 and 10.8 times, respectively, compared with women without the risk factor and not using oral contraceptives (*15*). Risk factors for stroke that are more potent or more prevalent among women include migraines with aura, atrial fibrillation, diabetes, depression, and psychosocial stress (*10*). Young women who experience migraines with aura have a twofold increase in the risk for stroke compared with women without migraines; for women with migraines with aura who also smoke and use oral contraceptives, the risk for stroke increases approximately seven times compared with women who do not smoke or use oral contraceptives (*16*). Depression and psychosocial stress increase the risk for stroke approximately 30% in both men and women (*17*).

## Impact of Stroke Systems of Care

When a stroke occurs, recognition and prompt treatment is critical. Each minute that an ischemic stroke is left untreated, the brain can lose nearly two million neurons (*18*). Emergency treatments that quickly return blood flow to the brain by dissolving or removing the clot blocking a brain artery have been found to substantially improve outcomes in ischemic stroke patients (*19*). Providing the right care at the right time is critical; one challenge to this is the fragmentation of stroke care. Currently, most acute stroke care is provided in distinct care delivery settings (e.g., emergency medical services [EMS], emergency department, hospital, and home or next care setting). With each transition, the potential for inefficient or suboptimal care and confusion for patients and their families can arise because of a lack of effective communication from one care setting and professional to the next. Viewing these elements as a stroke system of care, where the multiple, distinct components function as an efficient and effective integrated system, can overcome the fragmentation and reduce morbidity and mortality for acute stroke patients.

Developing stroke systems of care requires leadership and support from within each element to build working relationships across the system. Potential collaborators include state and local public health agencies; state, regional, and local EMS personnel; and clinical leaders. There are three critical functions of a well-integrated stroke system of care. First, stroke systems of care should establish effective interaction and collaboration. Integration across agencies, services, and people assures efficient routing of patients from the location of stroke occurrence to the closest and most appropriate level of hospital care in a locality or region to ensure timely access to treatment (*20*). Second, stroke systems of care should promote the use of an organized, standardized approach to stroke care at each facility and component within the system. Current practice for stroke systems of care is based in part on evidence-based recommendations from the Brain Attack Coalition (https://www.brainattackcoalition.org), including recommendations for different levels of stroke care (e.g., comprehensive stroke centers, primary stroke centers, and acute stroke ready hospitals) (*18*), and when appropriate, the use of telemedicine to provide timely acute stroke care to remote stroke treatment hospitals (*21*,*22*). Telemedicine for acute stroke care, or “telestroke,” allows stroke specialists to examine and communicate with potential stroke patients and physicians at remote hospitals using digital video technology, providing expert diagnosis and faster treatment for patients. Third, stroke systems of care must identify performance measures and outcomes that can be monitored to improve the quality of care provided. Through collaboration and use of the principles of continuous quality improvement, goals for the entire stroke system of care can be established to achieve better outcomes.

## Federal Programs to Prevent Stroke and Improve Stroke Care

The CDC’s Paul Coverdell National Acute Stroke Program (PCNASP) functions at the integration of clinical care and community-based public health. In 2001, PCNASP began funding academic principal investigators in eight states, and since 2004, has funded thirteen state health departments to improve the quality of care for acute stroke patients (*23*). The program works across the continuum of care to improve the quality of care provided, promote stroke prevention communication in communities, and improve transitions in care from EMS personnel to emergency department staff members and from hospital to rehabilitation and transition to home The goal of PCNASP is to implement effective, evidence-based, integrated systems for stroke prevention and treatment that provide 1) timely identification and transport of stroke patients to hospitals specializing in stroke care, 2) high-quality acute stroke treatment and rehabilitation, and 3) reintegration with primary care providers and the community to prevent recurrence of strokes by minimizing risk factors. From 2005 to 2015, approximately 620,000 acute stroke patients have benefited from care at PCNASP-participating hospitals, and each day many more patients are benefitting from the development of integrated stroke systems of care within PCNASP-funded states (*24*–*27*).

Since at least 2012, the National Institutes of Health has invested approximately $300 million annually to improve stroke prevention, treatment, and recovery (*28*). The National Institute of Neurologic Disorders and Stroke funds StrokeNet, a network, designed to maximize efficiencies in stroke research and to create balanced research in both preclinical and clinical trials. The National Institute of Neurologic Disorders and Stroke is also working to educate millions of Americans about the danger of uncontrolled hypertension through the Mind Your Risks campaign (https://mindyourrisks.nih.gov/).

The Million Hearts initiative (https://millionhearts.hhs.gov/), co-led by the CDC and the Centers for Medicare and Medicaid Services, focuses on a core set of strategies to prevent heart attacks and stroke. Community prevention includes tobacco control, sodium reduction, and physical activity. Clinical strategies include using aspirin when appropriate, hypertension control, cholesterol management, and smoking cessation, along with the use of health information technology to improve detection and management of patient-level risk factors for heart disease and stroke and patient and family engagement in health care decisions.

For strokes, prevention is the best medicine, whether the intervention is at the clinical or community level. Public health actions for stroke prevention include 1) employing epidemiology and surveillance to identify where progress is being made and where health care delivery gaps exist and to monitor and evaluate progress toward reducing those gaps; 2) promoting health system interventions to more effectively deliver high-quality preventive services; 3) improving community-clinical linkages to enhance access to community resources that can prevent, delay, and manage chronic diseases; and 4) implementing broad environmental approaches to improve the social and physical environment, promote healthy behaviors, and make healthy choices the easier choices (*29*). Collectively, these measures can prevent stroke.
